# Children’s soap pH analysis and its meaning in children’s skin cleansing

**DOI:** 10.1590/1984-0462/2025/43/2025035

**Published:** 2025-12-12

**Authors:** Rafaela Moura de Oliveira, Maria Carolina Schneider, Izabella Rodrigues Reis Gomes, Larissa Habib Mendonça Gois, Mariana Aparecida Pasa Morgan, Kerstin Taniguchi Abagge

**Affiliations:** a Universidade Federal do Paraná, Complexo Hospital de Clínicas, Postgraduate Program in Child and Adolescent Health, Curitiba, PR, Brazil.; b Universidade Federal do Paraná, Curitiba, PR, Brazil.; c Universidade Federal do Paraná, Department of Pediatrics, Pediatric Dermatology Division, Curitiba PR, Brazil.; d Faculdade Ceres, São José do Rio Preto, SP, Brazil.

**Keywords:** Analytical chemistry techniques, Skin, Skin care, Child health, Soaps, pH, Técnicas de análise química, Pele, Higiene da pele, Saúde infantil, Sabões, pH

## Abstract

**Objective:**

The aim of this study was to evaluate the surface hydrogen potential (pH) values of children’s soaps to identify those with a pH closer to the physiological one (5.0–5.5) and describe the most prevalent surfactants in the sample.

**Methods:**

This was a observational, analytical, cross-sectional, and quantitative study that included 96 cleansing products intended for children’s or sensitive skin, available at over-the-counter sales points in a Brazilian capital. The pH of the samples was measured after dilution to 1% in distilled water. A total of 34 bar soaps and 62 liquid soaps (including 20 syndets) were analyzed. Descriptive statistics and Student’s t-test were used to analyze the differences between the groups, with a minimum significance level of 5%. The surfactants present in the products were listed, and the most prevalent were identified.

**Results:**

The pH of the products ranged from 4.54 to 11.01, with a mean of 7.80±1.90. The pH levels differed between the liquid and bar formulations (4.54–8.00 vs. 8.01–11.01, respectively; p<0.001). The syndets had pH values closer to the physiological range (4.99–7.78), with lower values when compared to liquid soaps (p<0.001). The most prevalent surfactant in the sample was cocamidopropyl betaine.

**Conclusions:**

Bar soaps do not have a pH suitable for children’s skin cleansing, whereas liquid soaps, particularly syndets, have a pH closer to the physiological range. Knowledge of the pH values of children’s cleansing products by health professionals and caregivers is important, given the wide variety found in this study and the lack of reliable information on the packaging of most products.

## RESUMO

**Objetivo:**

Avaliar os valores de pH dos sabonetes infantis a fim de identificar aqueles com pH mais próximo ao fisiológico (5,0–5,5) e determinar os surfactantes mais prevalentes na amostra.

**Métodos:**

Estudo observacional, analítico, transversal e quantitativo com 96 produtos de limpeza destinados à pele infantil ou à pele sensível, encontrados em pontos de venda livre de uma capital brasileira. Procedeu-se à mensuração do pH das amostras após diluição a 1% em água destilada. Foram analisados 34 sabonetes em barra e 62, líquidos (sendo 20 *syndets*). Além da estatística descritiva, utilizou-se o teste *t* de Student para analisar as diferenças entre as médias de pH dos grupos, com nível de significância mínimo de 5%. Realizou-se, também, listagem e classificação dos surfactantes.

**Resultados:**

O pH variou de 4,54 a 11,01, com média de 7,80±1,90. Os níveis de pH diferiram entre as formulações líquida e em barra (respectivamente 4,54–8,00 vs. 8,01–11,01; p<0,001). Os *syndets* tiveram os valores mais próximos ao pH fisiológico (4,99–7,78), sendo os valores menores quando comparados aos demais sabonetes líquidos (p<0,001). O surfactante mais prevalente na amostra foi cocamidopropil betaína.

**Conclusões:**

Os sabonetes em barra não possuem pH adequado para cuidados da pele infantil, sendo os líquidos, em especial os *syndets*, os que apresentam pH mais próximo ao fisiológico. O conhecimento dos valores de pH dos produtos de limpeza infantil por profissionais de saúde e cuidadores é importante, dada a grande variedade encontrada neste estudo e a falta de informações confiáveis nas embalagens da maioria dos produtos.

## INTRODUCTION

The skin acts as a protective organ of the human body, and changes in its integrity increase the risk of infections, allergies, and thermal instability.^1,2^ The barrier function of the skin is represented by biophysical parameters such as surface hydrogen potential (pH), transepidermal water loss (TEWL), and stratum corneum (SC) hydration. These parameters undergo dynamic changes during the neonatal period.^2,3^

The final stages of structural and functional skin development, beginning in the third trimester of gestation, continue into the first months of life.^2^ At birth, the skin’s pH is almost neutral (around 7.5) but decreases to an acidic value (around 5.5) during the first weeks, resembling adult skin. Maturation of the acid mantle is essential for epidermal barrier homeostasis and is directly influenced by the cleanser used from the first bath of the newborn (NB).^2,3,4,5^

Over the years, the pharmaceutical industry has produced various cleansers with different compositions and skin-specific benefits. A key component of these cleansers is surfactants, which reduce surface tension between water and skin debris, facilitating their removal during bathing.^6^

Surfactants are classified based on their electrical charge into anionic, cationic (they have little cleansing action), non-ionic, or amphoteric, which influences the product’s pH and cleansing properties.^6^ Extensive studies on the interaction between surfactants and SC proteins have established a known order of irritation potential: anionic surfactants are more irritating than amphoteric, which in turn are more irritating than non-ionic surfactants.^7^

The development of syndet (synthetic detergents) products has improved the cosmetic acceptance and tolerability of skin cleansers.^8,9^ Syndets contain less than 10% detergent/surfactant and are pH-adjusted between 5.5 and 7 using synthetic detergents, resulting in effective cleansing with minimal lipid removal and irritation.^6^ They also cause less protein denaturation and restore lost moisturizing agents.^3,8^ Consequently, syndets are preferred for individuals with sensitive skin or dermatological conditions.^5^

In addition to soaps and syndets, a “combar” (combination bar) formulation combines both, offering more effective cleansing with less lipid damage compared to traditional soaps. Combars are widely available as bar and liquid soaps on the market.^8^

Given the importance of adapting children’s daily skin cleansers to conditions that cause skin sensitivity, the choice of soap can either support or threaten skin integrity. This depends on factors such as pH, surfactant concentration and quality, and the presence of other irritants or allergens like fragrances, preservatives, and dyes.

This study evaluated the pH levels of children’s soaps and syndets from retail stores in a capital city in southern Brazil, identifying products with pH levels closest to the physiological range (5.0–5.5). Additionally, the surfactants present in the samples were identified, with the most prevalent ones highlighted. This information aims to inform and update healthcare professionals and caregivers on the most appropriate products for children’s skin cleansing.

## METHOD

This is an observational, analytical, cross-sectional, quantitative study. Samples were obtained from all brands of children’s soaps (liquid or bar) and syndets found for sale in stores, supermarkets, and drugstores, located within a radius of 15 km from a university hospital in Southern Brazil, from May to September 2021. More than 10 points of sale were visited. In cases where the same product was identified at multiple points of sale, only a single sample was selected for laboratory analysis. The selection was based on the first point of purchase. A total of 96 soaps were found, among liquids, bars, and syndets. These were subdivided into five categories by pH ranges: lower than 5.0, between 5.0 and 5.9, between 6.0 and 6.9, between 7.0 and 7.9, and equal to or higher than 8.0.

This research did not require Ethics Committee approval because the data are publicly available and do not involve a direct interaction with human beings.

The pH of all soaps was measured once with a pH meter (Hanna^®^ Instruments, model HI 9321), calibrated with Alphatec Alpha 770 buffer solutions at pH 7.0 and 4.0, at 20°C. In the bar presentations, dilution was made at 1% in distilled water (pH=7.0). The data were stored in a Microsoft Excel^®^ spreadsheet. Variables were expressed as mean and standard deviation, median and confidence interval, as well as absolute and relative frequencies. The Student’s t-test was used to analyze the differences between the means of the groups (parametric): liquids vs. bars and liquids vs. syndets. All analyses were performed with the Statistic 10.0 Program (StatSoft^®^).

Information contained on the labels about the type of surfactants used, the pH value, and product descriptions such as “mild,” “hypoallergenic,” or “dermatologically tested” was evaluated. In addition, ingredients classified as “surfactants — cleansing agents” by the European Commission’s cosmetics industry database were cataloged in a Microsoft Excel^®^ spreadsheet, and the 10 most prevalent in the sample of liquid presentation products were listed.

## RESULTS

Of the 96 products evaluated, 42 were liquid soaps, 34 were bar soaps, and 20 were syndets. All syndets were in liquid form. The pH values of the products ranged from 4.54 to 11.01, with a mean of 7.80±1.90. Among the liquid soaps, the mean pH was 6.50±0.87, with values ranging from 4.54 to 8.08. Among the bar soaps, the mean was 10.25±0.80, with a pH range of 8.01–11.01. And the syndets had pH values ranging from 4.99 to 7.78, with a mean of 6.00±0.73.

Among the liquid soaps, 2 (4.8%) had pH <5.0, 7 (16.5%) between 5.0 and 5.9, 13 (30.9%) between 6.0 and 6.9, 18 (43.0%) between 7.0 and 7.9, and 2 (4.8%) >8.0 (Table 1). All bar soaps (100%) had pH >8.0 (Table 1). In the syndets’ group, 1 (5.0%) presented pH <5.0, 9 (45.0%) between 5.0 and 5.9, 8 (40.0%) between 6.0 and 6.9, and only 2 (10.0%) between 7.0 and 7.9 (Table 1).

**Table 1:** Trade name and pH of the soaps evaluated in ascending order by category, liquid, bar and syndets.

*Liquid soaps*		*Bar soaps*		*Syndets*	
Soaps	pH	Soaps	pH	Soaps	pH
**L1**	4,54	**B1**	8,01	**S1**	4,99
**L2**	4,59	**B2**	8,07	**S2**	5,26
**L3**	5,47	**B3**	8,19	**S3**	5,28
**L4**	5,47	**B4**	8,36	**S4**	5,3
**L5**	5,5	**B5**	9,82	**S5**	5,53
**L6**	5,54	**B6**	9,99	**S6**	5,61
**L7**	5,8	**B7**	10,17	**S7**	5,79
**L8**	5,86	**B8**	10,27	**S8**	5,83
**L9**	5,92	**B9**	10,29	**S9**	5,87
**L10**	6,14	**B10**	10,31	**S10**	5,91
**L11**	6,31	**B11**	10,37	**S11**	6,02
**L12**	6,34	**B12**	10,4	**S12**	6,07
**L13**	6,38	**B13**	10,42	**S13**	6,1
**L14**	6,44	**B14**	10,43	**S14**	6,13
**L15**	6,6	**B15**	10,43	**S15**	6,25
**L16**	6,66	**B16**	10,49	**S16**	6,28
**L17**	6,7	**B17**	10,51	**S17**	6,92
**L18**	6,84	**B18**	10,52	**S18**	6,95
**L19**	6,86	**B19**	10,58	**S19**	7,5
**L20**	6,88	**B20**	10,63	**S20**	7,78
**L21**	6,97	**B21**	10,65		
**L22**	6,98	**B22**	10,65		
**L23**	7,02	**B23**	10,66		
**L24**	7,05	**B24**	10,66		
**L25**	7,06	**B25**	10,68		
**L26**	7,07	**B26**	10,68		
**L27**	7,08	**B27**	10,68		
**L28**	7,1	**B28**	10,69		
**L29**	7,21	**B29**	10,72		
**L30**	7,25	**B30**	10,72		
**L31**	7,37	**B31**	10,86		
**L32**	7,38	**B32**	10,93		
**L33**	7,39	**B33**	10,95		
**L34**	7,43	**B34**	11,01		
**L35**	7,51				
**L36**	7,53				
**L37**	7,55				
**L38**	7,55				
**L39**	7,81				
**L40**	7,92				
**L41**	8				
**L42**	8,08				

**Figure 1:**
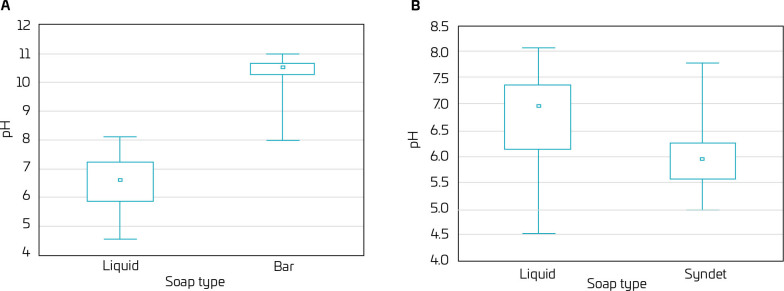
pH variation according to soap type. (A) Liquid vs. bar; n=76. (B) Liquid vs. syndets; n=62.

Legend: Student’s t-test: p<0.001 for both comparisons.

When comparing the pH values between liquid and bar soaps, liquid soaps showed significantly lower pH values (p<0.001) (Figure 1A). Among liquid soaps and syndets, syndets showed lower pH values (p<0.001) (Figure 1B).

Among all the products evaluated, 31 (32.3%) had information about pH on the label. Among the liquid soaps, 18 (42.8%) contained information such as “balanced pH,” “neutral pH,” “physiological pH,” “skin pH,” “balanced pH,” and “ideal pH.” One of them specifically exhibited “pH 7,” with a pH measurement result of 7.39. Among the syndets, 8 (40.0%) mentioned information about pH. Among the bar soaps, three soaps from the same brand had the information “pH neutral” on the package, and two others had “maintains the skin’s natural pH,” totaling 14.7% of the sample.

All the liquid soaps and syndets (100%) and 31 of the bar soaps (91.1%), display claims on their labels such as “dermatologically tested,” “hypoallergenic,” “tested by pediatricians,” “formulated to minimize the risk of allergic reactions,” and “delicate protection.” The most prevalent information was “dermatologically tested.”

Regarding the surfactants cataloged, 80 different surfactants were identified, and the 10 most prevalent are listed in Table 2. The most prevalent surfactant in the sample was cocamidopropyl betaine, found in 43 products.

**Table 2:** Most prevalent surfactants in the composition of liquid soaps and syndets.

Surfactant	n (%)
Cocamidopropyl betaine	43 (69.4)
Sodium lauryl ether (laureth) sulfate	22 (35.5)
Decyl glucoside	17 (27.4)
Peg-150 distearate	13 (21.0)
Lauryl glucoside	12 (19.4)
Cocamide DEA	11 (17.7)
Coco-glucoside	11 (17.7)
Disodium cocoyl glutamate	9 (14.5)
Disodium lauryl ether (laureth) sulfosuccinate	8 (12.9)
Sodium trideceth sulfate	7 (11.3)

## DISCUSSION

This study evaluated the pH of 96 infant cleansers and revealed that the pH of syndet soaps (4.99–7.78) was closer to the physiological pH of the skin (5–5.5). Despite being specifically designed for sensitive and atopic skin, two of these products had a pH value higher than 7.

When comparing liquid and bar soaps, the liquid presentation products had a pH closer to physiological pH (4.54–8.0), while the bar soaps had the highest pH values (8.01–11.01) (p<0.001). Only a minority of products (32.3%) disclosed specific pH information on their labels.

The quality of the surfactant used in cleansing products is another important factor to be considered in their choice.^10^ In this survey, the most prevalent surfactant in the sample was cocamidopropyl betaine, an amphoteric surfactant, a group widely used in rinse-off personal care products. It is characterized by good cleansing action and foaming ability, moderate antimicrobial activity, and compatibility with different pHs. Cocamidopropyl betaine has a low association with irritation and allergic contact dermatitis, reactions that may be due to the ingredient itself or to impurities present in it.^11,12^

One limitation of this study is that it focused exclusively on analyzing the pH and surfactant types of infant cleansing products, without assessing other important formulation components such as preservatives, fragrances, dyes, and potential endocrine-disrupting chemicals.^12^ These additional ingredients may also influence skin tolerance and safety and should be considered in future research. Another limitation was the number of establishments visited. For a more representative sample of a city with almost two million residents, the ideal number of points of sale would be close to 100, distributed across the 9 districts of the municipality. Even so, given that the main products and brands are evenly distributed throughout the city, we consider that the present study is valid in representing the products most frequently used by the population.

These findings underscore the importance of selecting cleansing products that align with the skin’s physiological pH to minimize potential irritation and support skin barrier function. The results provide valuable insights for healthcare professionals and caregivers, assisting in informed decision-making regarding pediatric skincare.

## Data Availability

The database that originated the article is available with the corresponding author.
